# Conformational Flexibility of GRB2 as a Key Factor in the Stability and Regulation of Its Interaction with SOS1

**DOI:** 10.1021/acsomega.5c01677

**Published:** 2025-06-30

**Authors:** Renan P. Pedro, Raphael V. R. Dias, Ingrid B. S. Martins, Murilo Nogueira Sanches, João V. Piloto, Icaro P. Caruso, Vitor B. P. Leite, Fernando A. de Melo

**Affiliations:** † Department of Physics, Institute of Biosciences, Humanities and Exact Sciences, 135131 São Paulo State University (UNESP), São José do Rio Preto, SP 15054-000, Brazil; ‡ Institute of Chemistry, São Paulo State University (UNESP), Araraquara, SP 14800-060, Brazil

## Abstract

The dysregulated activation of the Ras pathway is directly associated with approximately one-third of human cancer cases. The interaction between the adaptor protein GRB2 and the guanine nucleotide exchange factor SOS1 plays a crucial role in this process, serving as a key link in the activation of Ras. This interaction facilitates signal transduction that regulates essential cellular processes such as proliferation, survival, and differentiation and is, therefore, a central point in oncogenesis. Although the GRB2-SOS1 complex is critical for the regulation of Ras pathway signaling, the structural mechanisms governing this interaction remain largely unknown. In this study, we conducted an in-depth analysis of the interaction between GRB2 and peptides derived from SOS1, utilizing computational modeling, docking, and molecular dynamics simulations, combined with a detailed analysis of the energy landscape (ELViM). The results demonstrate that the conformational flexibility of the GRB2 protein has a direct impact on the stability of the GRB2-SOS1 complex, with communication between GRB2 domains being a determining factor for the robustness of this interaction. Additionally, we identified critical residues that play decisive roles in the formation and regulation of this interaction, which may serve as potential targets for modulation of the Ras pathway. These findings not only deepen the understanding of the structural mechanisms underlying the GRB2–SOS1 interaction but also provide a foundation for the development of specific therapeutic strategies aimed at controlling aberrant Ras pathway signaling, thereby offering new prospects in the treatment of diseases associated with the anomalous activation of Ras.

## Graphical abstract

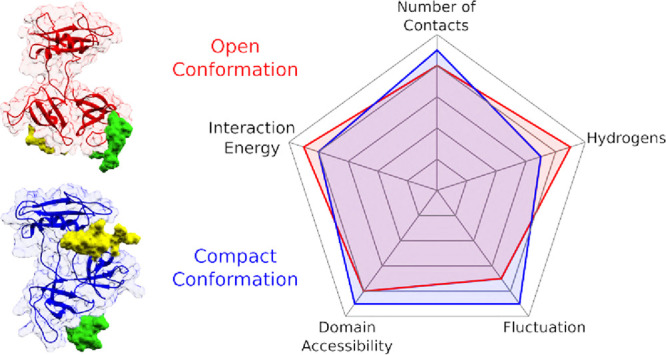

## Introduction

The Growth Factor Receptor-Bound Protein 2 (GRB2) is a key adaptor protein in the regulation of intracellular signaling. It establishes a vital connection between the Receptor Tyrosine Kinase (RTK) and guanine nucleotide exchange factors specific for Ras (RasGEFs), which is fundamental to the cellular growth response, triggering a cascade of molecular events.^1–5^ In situations of cellular growth stimulation, such as through the Epidermal Growth Factor Receptor (EGFR) or other prominent RTKs such as the Fibroblast Growth Factor Receptor (FGFR), phosphorylation of the kinase domain occurs.^1,6–8^ In this context, the RTK, in association with a dimeric configuration of GRB2, induces GRB2 phosphorylation, resulting in the release of the autoinhibited GRB2 homodimer.^1,7,9^

The active monomeric form of GRB2 plays a crucial role in recruiting Son of Sevenless 1 (SOS1), a guanine nucleotide exchange factor specific for Ras (RasGEF), toward the cell membrane. SOS1 is essential for facilitating the exchange of GDP for GTP in the small Ras GTPase, triggering a series of biochemical events that direct cell proliferation.^2,4,10,11^ Subsequent activation of Ras GTPase initiates an intracellular signaling cascade, featuring two main pathways: the Mitogen-Activated Protein Kinase (MAPK, Raf/MEK/ERK) pathway and the Phosphatidylinositol 3-Kinase (PI3K)/Akt/mTOR pathway.^12–14^ Both pathways play crucial roles in cell cycle regulation and the promotion of cell proliferation. It is crucial to emphasize that oncogenic mutations in three specific Ras isoforms are directly associated with approximately one-third of human somatic cancers.^13,15^

Given the complexity of targeting oncogenic Ras, scientific research focuses on identifying inhibition strategies, both upstream, involving components like SOS1, GRB2, EGFR, and FGFR, and downstream, encompassing targets like Raf and PI3Kα.^16–19^ In this context, it is pertinent to highlight that the interaction between GRB2 and SOS1 has been the subject of notable emphasis in clinical contexts.^20,21^ However, a significant gap persists in the molecular understanding of the mechanisms underlying this interaction.

The GRB2 protein’s structure comprises three Src homology (SH) domains: one SH2 domain situated between two SH3 domains^1^ (Figure 1A). The N-terminal SH3 domain (NSH3, residues 1–58) and the C-terminal SH3 domain (CSH3, residues 155–217) are positioned at the ends of the SH2 domain (residues 59–154) (Figure 1C).^23^ The SH2 domain is involved in the recognition of phosphorylated tyrosine (pY) residues originating from receptor tyrosine kinases (RTKs), while the NSH3/CSH3 domains are pivotal in accommodating the proline-rich (PR) domain of the SOS1 protein (residues 1014–1333) (Figure 1D).^24–28^

**Figure 1:**
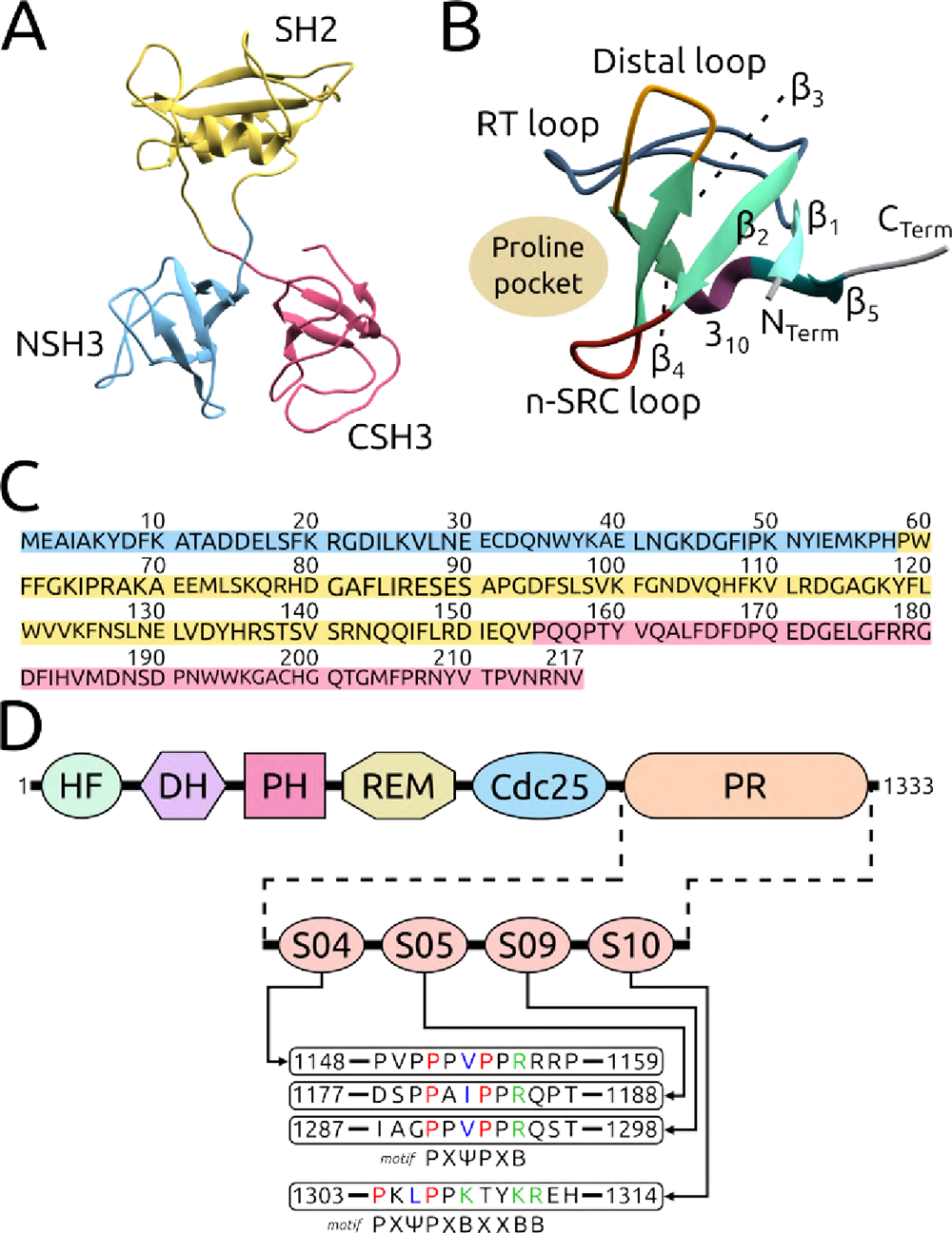
Structural organization and sequence analysis of GRB2 and SOS1. (A) Complete structure of the human GRB2 protein (PDB ID 1GRI,^22^ consisting of 217 amino acid residues. The protein is composed of the NSH3 domain (blue, residues 1–58), SH2 domain (yellow, residues 59–154), and CSH3 domain (pink, residues 155–217). (B) Representation of the NSH3 domain, highlighting the general structure typical of an SH3 domain, including its β-strands (shades of green) and the small 3_10_-helix (claret). The RT, n-SRC, and Distal loops form a specific interaction site that recognizes proline-rich regions in partner proteins. (C) Primary structure of the GRB2 protein, with regions comprising the NSH3, SH2, and CSH3 domains highlighted in the same colors used in (A). (D) Proline-rich domain (PR) of SOS1, located at the C-terminal end of the protein. This PR domain contains four distinct sites (designated S04, S05, S09, and S10), characterized by the motifs PxΨPxB or PxΨPxBxxBB, where P represents proline residues, X any residue, Ψ hydrophobic residues, and B basic residues. The complete sequences of these regions are presented. Other domains of SOS1 shown include HF (histone fold), DH (Dbl homology), PH (pleckstrin homology), REM (Ras exchange motif), and Cdc25.

The SH2 domain adopts a conformation of a globular module characterized by a highly conserved secondary structure, consisting of a central antiparallel β-sheet surrounded by two α-helices at the ends.^29^ The SH3 domains constitute protein modules with a secondary structure characterized by a small 3_10_-helix, along with four to eight β-strands arranged in two β-sheets or a β-barrel.^30^ Additionally, the loops of the domain are designated as RT, n-Src, and distal, representing crucial regions in partner recognition for molecular interaction (Figure 1B).^30,31^ Previous research has indicated that the SH3 domain exhibits an affinity for PxxP interaction motifs, occupying two distinct sites. Peptides containing the PxxP motif can interact through two opposite conformations, determined by the relative position of a positively charged residue (+xxPxxP or xPxxPx+).^26,31^ These conformations are essential for how the domain establishes interactions with other proteins.

SOS1 has four distinct sites in its proline-rich domain (PR) that bind to the SH3 domains of GRB2 (Figure 1D).^32^ These sites, designated S04, S05, S09, and S10, follow the consensus motif PxΨPxB or PxΨPxBxxBB, where X represents any residue, Ψ corresponds to valine, leucine, or isoleucine (hydrophobic residues), and B is lysine or arginine (basic residues).^24–26^

The formation of the GRB2-SOS1 complex was first described in the early 1990s.^33,34^ Since then, various studies have investigated the interaction between GRB2 and multiple SOS1 peptides in detail.^24,26,35–37^ These studies have demonstrated that the NSH3 domain of GRB2 is typically the primary site for binding to SOS1, while the CSH3 domain contributes to the stability of the complex.^25^ It also highlighted the complex structural flexibility of GRB2 due to its role as an adaptor protein,^38–42^ which allow GRB2 to to access various conformational states, particularly in its monomeric form.^23^ The importance of interdomain communication within GRB2 for interactions with other molecules and its structural formation, including its folding process, has also been reported.^23,39,41,43^ However, these observations lack detailed molecular explanations and rely mainly on the crystal structure (PDB ID 1GRI),^22^ which is the only experimentally resolved structure of the complete protein and is in a dimeric state, potentially not reflecting the reality of the interactions, since GRB2 in its dimeric form inhibits SOS1 binding.^7^ Furthermore, the specific molecular mechanisms that governs the interaction between GRB2 and SOS1, particularly within the complete GRB2 protein structure, remain unclear. Most studies focus only on isolated domains, raising questions about the interaction mechanism and behavior when considering the full-length protein.

Considering this, we aimed in this study to understand the GRB2/SOS1 interaction, focusing this time not on the effects of the GRB2 protein on SOS1 (or its peptides), but on the changes that occur in GRB2 during this interaction. Our goal was to identify which residues of the GRB2 protein are directly and indirectly responsible for the interaction, as well as to observe whether conformational changes in GRB2 would affect this interaction.

In this study, we used four distinct peptides derived from SOS1 (Figure 1), of which three interact with the NSH3 domain and two with the CSH3 domain. The analyses were conducted considering the average of these interactions, as the pairs of complexes (protein/peptide and domain/peptide) exhibited similar behaviors.

We performed molecular docking studies and Molecular Dynamics (MD) simulations of the monomer and the isolated NSH3 and CSH3 domains of GRB2 (PDB ID: 1GRI^22^) in complex with SOS1 peptides, resulting in a comprehensive investigation of the molecular interactions between these proteins. Initially, the MD simulations of the GRB2 monomer revealed two predominant conformations: a more open state, similar to that observed in crystalline structures, and a more closed state, where the CSH3 domain approaches the SH2 domain (see section Monomer-only Molecular Dynamics). This latter conformation has been reported in the literature as a potential alternative structure. Consequently, the protein-peptide complexes were analyzed in both conformations. The results of the simulations of the isolated domains interacting with the peptides showed that the NSH3 domain exhibited greater stabilization when interacting with the peptides. When the full protein interacts with the peptides, the data support this observation, indicating stabilization of the NSH3 domain and suggesting a cooperative interaction involving the entire protein, including the SH2 domain. Furthermore, our results identified key residues involved in the interactions within the NSH3 and CSH3 domains, enhancing the understanding of the assembly of a critical signaling complex for the cellular machinery by providing molecular details on how the GRB2 protein interacts with various segments of SOS1-PR. These findings could be fundamental for the development of peptidic inhibitors aimed at blocking the GRB2-SOS1 interaction, thereby inhibiting RAS activation.

## Methods

### System Preparation

The structure of the GRB2 protein was initially obtained from the Protein Data Bank (PDB ID 1GRI, chain A^22^). However, it is worth noting that the protein in this reference had gaps, necessitating the use of molecular modeling through the I-TASSER server^44^ for its completion. Once the complete version of the protein was obtained, a molecular dynamics simulation was carried out exclusively for the protein. This simulation revealed two possible conformations for the GRB2 protein.

One conformation resembles the structure obtained via crystallography, while the other exhibits a more compact conformation, with the SH2 domain close to the CSH3 domain. The choice between these two structures was based on the assumption of equal probability of occurrence for both, supported by literature reports.^22,38^ Therefore, the simulation of the GRB2/peptide interaction was conducted by using both structural models of GRB2 (see section Monomer-only Molecular Dynamics). The model derived from the crystal structure was termed Model01 (open state), while the more compact model was designated as Model02 (compact conformation). Regarding the complexes formed by NSH3/peptides and CSH3/peptides, the SH3 domains were derived from the GRB2Model01 structure (it is noteworthy that the SH3 domains remained stable in both Model01 and Model02, making it indifferent which structure to choose for isolating these domains), following the known amino acid sequence for each domain.^23^

### Molecular Dynamics

Molecular Dynamics simulations were conducted using GROMACS 2020,^45^ employing the AMBER99SB-ILDN force field.^46^ This force field was chosen due to its well-documented accuracy in modeling protein and peptide interactions, particularly its optimized parameters for side-chain torsion angles and improved performance in capturing secondary structure conformations, as validated in numerous studies.^47–49^

Preparation of protein/peptide complexes was carried out using the pdb 2gmx module, and these complexes were subsequently designated as initial points for simulations. All systems were solvated with the TIP3P water model, contained within a truncated cube with a 10 Å cutoff between the complexes and the box boundary during simulations. Sodium ions (Na^+^) and chloride ions (Cl^–^) were added to maintain electrical neutrality using the “gmx genion” script in GROMACS.^45^

Subsequently, position-restrained dynamics simulations, specifically NVT and NPT ensembles simulations (*N*, number of particles; *P*, system pressure; *V*, volume; and *T*, temperature), to equilibrate the systems, solvent, and ions surrounding the protein. A 500 ps NVT simulation was performed for each system, during which the systems were heated to 298 K (using the V-rescale thermostat^50^) with a coupling constant of 0.1 ps, aiming to simulate and establish the proper orientation relative to the solute. The NPT simulation (500 ps) of the systems was conducted, balancing the systems with a constant pressure of 1 bar and a coupling constant of 2 ps, using the leapfrog integrator to stabilize the system pressure and density, again at 298 K. This temperature was chosen to ensure consistency with the experimental conditions reported by Liao et al.^24^

The Particle Mesh Ewald (PME) algorithm was employed to calculate long-range electrostatic interactions.^51^ Short-range electrostatic interactions and van der Waals (VDW) interactions were truncated with a 1.0 nm radius throughout the simulations. All bonds, including hydrogen atoms, were constrained by the LINCS algorithm. Finally, all systems underwent a 500 ns molecular dynamics simulation, recording all trajectories every 100 ps, with a time step of 2.0 fs. All simulations were performed in triplicate, and the resulting data were analyzed by calculating the mean and standard deviation.

### Energy Landscape Visualization Method

The Energy Landscape Visualization Method (ELViM) was employed to identify and analyze the conformational ensembles sampled from the MD trajectories. ELViM is a multidimensional projection method designed to create intuitive representations of high-dimensional phase spaces in biomolecular contexts.^52^ Using an internal distance metric that describes the differences between the sampled structures, it maps a point onto a plane for each conformation, aiming to ensure that the pairwise Euclidean distances between points closely approximate the original dissimilarity among the conformations. A full description of the method and how to implement it is available on GitHub (https://github.com/VLeiteGroup/ELViM). ELViM has been previously applied to analyze domain rearrangements,^53^ as well as it was also applied to GRB2 to understand its monomeric folding.^23^

### Peptide’s Structure Prediction

Four peptide sequences were chosen from the ref 24 based on their biological relevance and potential functional activity. The selected sequences were S04 (PVPPPVPPRRRP), S05 (DSPPAIPPRQPT), S09 (IAGPPVPPRQST), and S10 (PKLPPKTYKREH). To predict the three-dimensional structures of these peptides, we used the PEP-FOLD server.^54^ The amino acid sequences were input to the PEP-FOLD server according to the provided instructions, ensuring the correct specification of parameters and available options. After downloading the prediction results, we proceeded with the analysis of stability and three-dimensional conformation. Details related to secondary structures, foldings, and areas potentially relevant to biological activity were meticulously considered. The predicted structures were then compared with those obtained in the ref 24, consolidating the analysis of the predictions.

### Docking

The process of peptide binding to SH3 domains and the GRB2 protein was simulated using the HADDOCK server.^55^ In the execution of the HADDOCK simulations, the experimental data previously obtained by Liao et al*.* ^24^ were adopted as a reference. Protein residues directly affected by interactions with peptides were designated as ‘active residues’, while those adjacent to them were termed ‘passive residues’, The interaction between active and passive residues constitutes the protein/peptide interface. The HADDOCK coupling protocol consisted of three consecutive stages. Initially, the molecules were randomly oriented, followed by a rigid-body energy minimization. The best-ranked models were then subjected to a semiflexible simulated annealing stage, performed in the torsion angle space. Finally, the structures obtained after the semiflexible simulated annealing were refined in an explicit solvent layer, aiming to further enhance their scores. The protein/peptide complexes with the lowest energies resulting from this process were selected for molecular dynamics simulations, representing the most stable and energetically favorable interactions without relying on structural templates or experimental constraints, enabling unbiased exploration of binding poses based purely on computational scoring functions.

### MM/GBSA Approach

The theoretical Gibbs free energy of binding contributions from the residues of GRB2 interacting with ligands was calculated using the Molecular Mechanics Generalized Born Surface Area (MM/GBSA) method. This method was implemented in the gmx_mmpbsa program^56^ for the GROMACS package, using igb = 2, GROMACS version 2020,^45^ and the AMBER99SB-ILDN force field.^46^ The calculations were based on 100 frames, using the last 400 ns of the simulation. The parameters used included a solute dielectric constant of 2, a solvent dielectric constant of 80, a NaCl concentration of 150 mM, and a temperature of 298 K.

## Results and Discussion

### Selection of Key Peptides

The results obtained in this study were influenced by the findings of Liao et al.,^24^ who investigated the interaction between the SH3 domains of the GRB2 protein (both N- and C-terminal) and peptides derived from the SOS1 protein using nuclear magnetic resonance (NMR). In Liao’s study, ten peptide sequences, designated S1 through S10, were analyzed. In this work, we highlight four peptides of significant relevance identified by Liao et al.,^24^ as presented in Table 1. Peptides S04, S05, S09, and S10 were selected based on the highest observed Δδ_MAX_ values, corresponding to the maximum chemical shift perturbation. These values, determined through NMR experiments, indicated that these peptides exhibited the most significant perturbations. Therefore, peptides S04, S05, S09, and S10 were considered the most notable in terms of their responses in the NMR experiments conducted by Liao et al.^24^ Among these peptides, S04, S05, and S09 interacted with the NSH3 domain, while S04 and S10 interacted with the CSH3 domain.

**Table 1:** Peptides Derived from the SOS1 Protein and Its Corresponding Associated Domains

name	residues	sequence	motif	domain GRB2
S04	1148–1159	PVPPPVPPRRRP	PxxPxR	NSH3 and CSH3
S05	1177–1188	DSPPAIPPRQPT	PxxPxR	NSH3
S09	1287–1298	IAGPPVPPRQST	PxxPxR	NSH3
S10	1303–1314	PKLPPKTYKREH	PxxPxKxxKR	CSH3

Unlike the study by Liao et al.,^24^ we extended our analysis beyond the SH3 domains, conducting simulations on the complete GRB2 protein to better understand how these specific interactions influence the overall structure and dynamics of the protein. We performed simulations of the peptides in both unbound and bound states, followed by clustering analyses (Figure S1) to identify the most representative conformations. Subsequently, we grouped the clusters into representative populations (Figure S2), allowing us to assess the structural stability of the different peptides. Overall, peptides S04 and S10 exhibited good stability in the unbound state, with even greater stability in the bound state. In contrast, peptides S05 and S09 displayed low stability in the unbound state but demonstrated high stability when interacting with the GRB2 protein in the bound state.

### Analysis of SH3/Peptide Complexes

In order to investigate the interaction between SH3 domains (NSH3 and CSH3) and peptides derived from SOS1, molecular dynamics simulations lasting 500 ns were performed using GROMACS 2020 software,^45^ with the AMBER99SB-ILDN force field.^46^ The details of the complexes are presented in Table S1, as described by Liao et al.^24^ Complex stability was evaluated through RMSD calculations, as depicted in Figure S3. The RMSD values calculated for the backbone of the NSH3 domains are presented in Figure S3A,B for the unbound and bound forms, respectively, for each peptide. The unbound form refers to the state of the protein or domain when it is not interacting with any peptide, while the unbound form indicates the state when the protein or domain is interacting with a peptide. Notably, both domains exhibit lower RMSD values in their bound forms compared to their unbound forms, suggesting stabilization of the domains during interactions with SOS1 peptides.

We also calculated the RMSF to quantify the fluctuation of atomic coordinates of residues, as well as the difference in RMSF values (ΔRMSF) between the unbound and bound forms. The ΔRMSF values were obtained by first calculating the RMSF of each SH3 domain in complex with each individual SOS1 peptide listed in the previous section. For each complex, RMSF values were computed across three independent molecular dynamics replicas and then averaged. These averaged values were subsequently combined across all peptides to generate a single ΔRMSF profile for each SH3 domain. This approach captures the overall conformational impact of peptide binding on SH3 domains, rather than emphasizing the effects of any single peptide interaction. The results are presented in Figure 2A. Therefore, the ΔRMSF values reflect the general behavior of the SH3 domains when interacting with any of the peptides, regardless of their sequence. We also provide a representation of residues that showed the greatest discrepancies between bound and free conformations in Figure 2B. Upon examining the NSH3 domain (Figure 2A), we observed that, in general, all residues become more stable when interacting with peptides (S05, S09, and S10) with exception of residues (Asn51 e Pro57) (ΔRMSF < 0). However, it is noteworthy that residues Asp14, Asp15, Asn29, Tyr37, Ala39, Glu40, Leu41 and Asn42 (Table 2) exhibit a more pronounced reduction in fluctuation, suggesting a more specific interaction with peptides. These residues are part of the RT-Src loop and the n-Src loop, as well as components of β3, regions of crucial relevance for the GRB2-SOS1 complex interaction.

**Figure 2:**
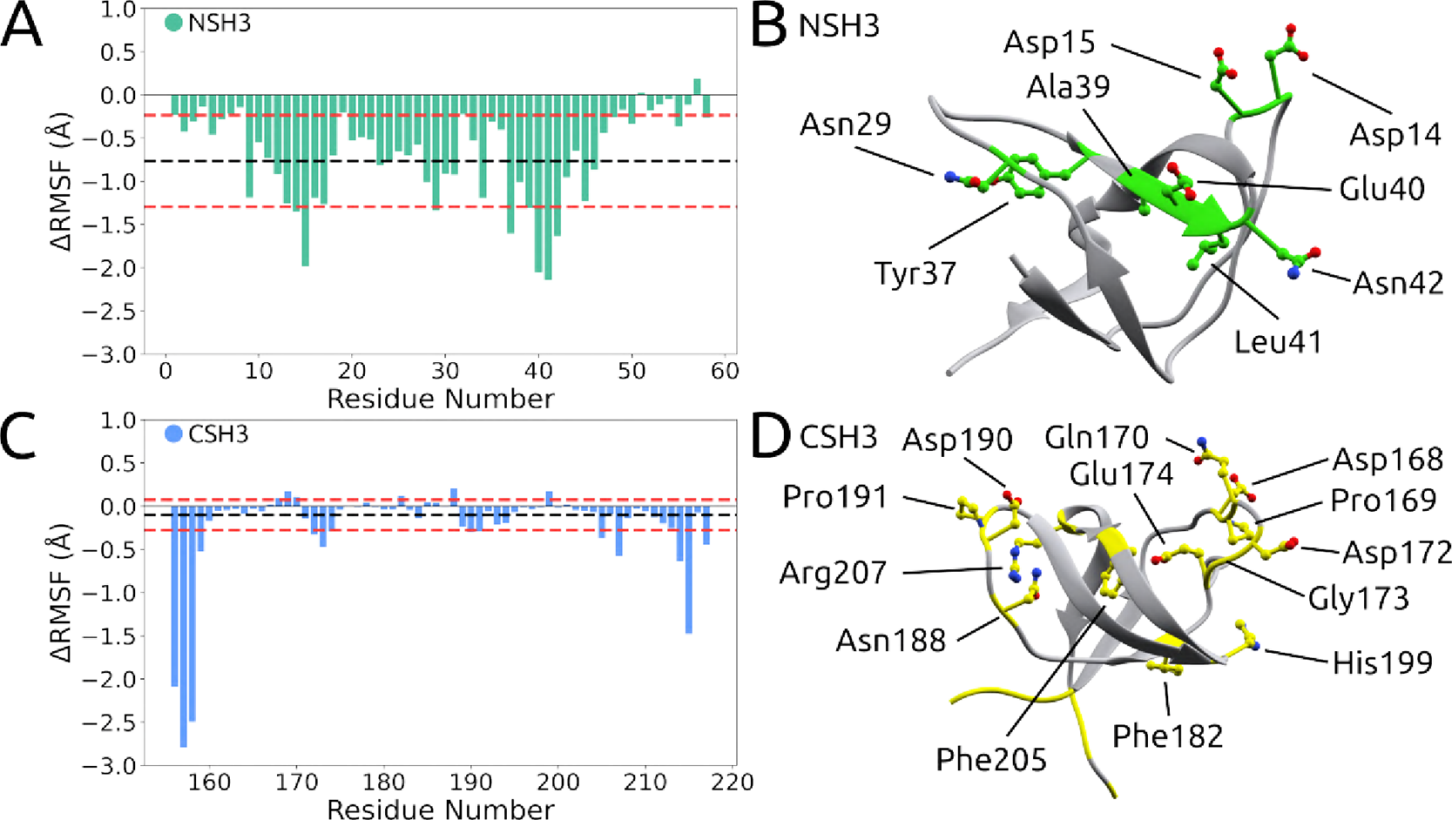
ΔRMSF and tertiary structure of SH3 domains. (A) Analysis of RMSF of the NSH3 domain interaction with peptides compared to its free conformation. The black dashed line denotes the RMSF mean, while the red dashed line indicates the standard deviation from this mean. (B) Cartoon representation of the NSH3 domain structure (PDB ID 1GRI^22^ highlights, in green shading, the residues whose ΔRMSF values are below the mean minus the standard deviation, as shown in (A). This indicates that these residues experienced the most significant changes upon interaction with the peptides. (C) Analysis of RMSF of the CSH3 domain in interaction with peptides contrasted with its uncomplexed conformation. The black dashed line represents the RMSF mean, while the red dashed line highlights the standard deviation from this mean. (D) Cartoon representation of the CSH3 domain (PDB ID 1GRI^22^) highlights, in yellow shading, the residues with ΔRMSF values below the mean minus one standard deviation, as well as those exhibiting ΔRMSF values above the mean plus one standard deviation, as observed in figure (C).

**Table 2:** Comparison of RMSF Differences between NSH3 and CSH3

	NSH3			CSH3		
ΔRMSF > 0	Asn51	Pro57		Phe167	Arg178	Val185
				Met186	Gln201	
ΔRMSF > AVG + SD	Met1	Ile4	Tyr7	Asp168	Pro169	Gln170
	Asp8	Phe19	Cys32	Phe182	Asn188	His199
	Pro49	Tyr52	Ile53			
	Glu54	Lys56				
ΔRMSF < AVG – SD	Asp14	Asp15	Asn29	Gln156	Gln157	Pro158
	Tyr37	Ala39	Glu40	Thr159	Asp172	Gly173
	Leu41	Asn42		Glu174	Asp190	Pro191
				Phe205	Arg207	Asn214
				Arg215	Val217	

Upon analyzing the CSH3 domain (Figure 2C), it is observed that there are few residues (Table 2) in the positive portion (ΔRMSF > 0), However, these residues exhibit ΔRMSF values between 0 and 0.01 Å, characterizing an insignificant perturbation to prevent the interaction. Further, an assorted number of residues presented ΔRMSF < 0 (Figure 2D), indicating that, overall, the CSH3 domain becomes more stable with the interaction. Nonetheless, the stability of the CSH3 domain is less affected compared to the NSH3 domain, suggesting that, although the interaction occurs in both domains, NSH3 is more notably affected. This analysis reveals that the NSH3 domain is of greater importance in mediating the interaction between GRB2 and SOS1.

To corroborate these findings and enhance the robustness of our analysis, we also calculated the prevalence of hydrogen bonds between the domains and peptides throughout the simulation. These results are presented in Figure 3. For the NSH3 domain, the peptide that presented the highest number of hydrogen bonds with the domain (occupancy higher than 20%) was S05, with five hydrogen bonds, followed by S04 with three hydrogen bonds and S09 with two hydrogen bonds. For the CSH3 domain, peptides S04 and S10 presented four hydrogen bonds each using the same metrics, corroborating the more probable mode of interaction for the GRB2 monomer suggested by Liao et al.^24^ These findings reveal the relation between the conformational dynamics of the CSH3 domain with its RMSF fluctuation, which is influenced by its interaction with the SOS1 protein. Specifically, the CSH3 domain maintains its overall conformation (as shown by the ΔRMSF values approaching zero between residues 160 and 213 in Figure 2C) when binding to different segments of SOS1. This stability indicates that CSH3 has an intrinsic local flexibility in this region, with the hydrogen bond occupancy (Figure 3) further supporting this analysis, showing that residues exhibiting stabilized dynamics also participate in persistent hydrogen bonds with SOS1-derived peptides.

**Figure 3:**
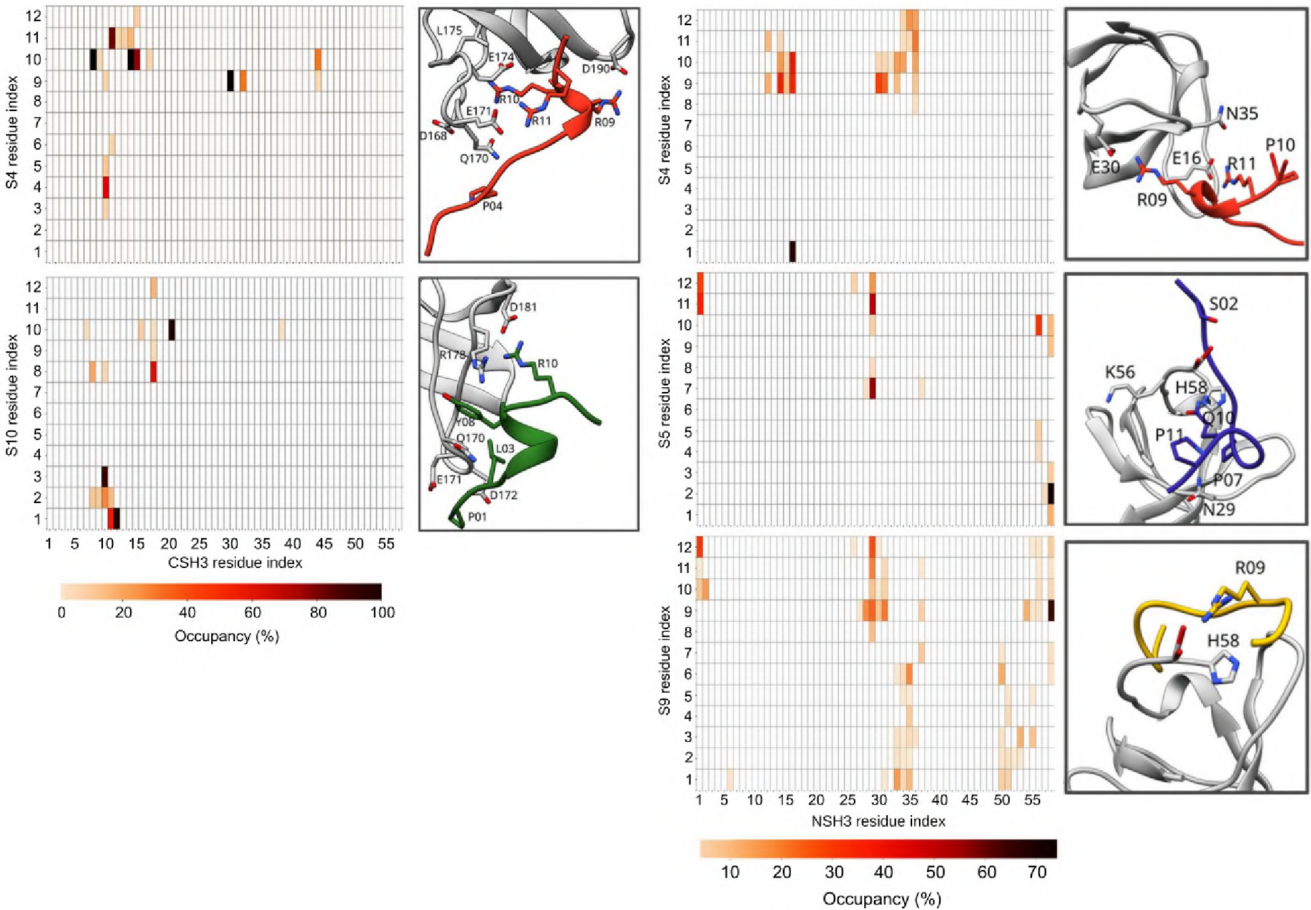
Hydrogen bonds of isolated SH3 domains with peptides. We present here an interaction map between GRB2 domains and SOS1 peptides. Each rectangle represents an interaction and is colored based on the prevalence of that interaction. The darker the rectangle, the longer the residues interacted during molecular dynamics (MD). We also highlight the main residues of each domain in the panels to the right of each map. It is important to note that we considered prevalence greater than 20% in the last 200 ns of the simulation.

### Monomer-Only Molecular Dynamics

Investigating the interaction between GRB2 and SOS1 peptides within the context of isolated domains has already been reported in other studies in the literature and was reinforced by us in the previous section.^24,25^ Here, we leverage these findings to explore the interaction within the full-length GRB2 protein in its monomeric form.

First, we performed a conventional molecular dynamics (MD) simulation of 1 microsecond (1 μs) with GRB2 (PDB ID 1GRI^22^). It is noteworthy that this crystal structure was resolved in its dimeric form; therefore, we used chain A to proceed with the MD simulation. We then employed the ELViM methodology to identify the relevant structures sampled in the MD. With this analysis, each conformation is represented in 2D by a dot, with the Euclidean distance between these dots corresponding to the structural similarity between the structures. Figure 4A displays the density of states for this 2D representation, which shows that there are two major conformations that were more recurrent during the MD. The first conformation (open state) is a structure similar to the crystallographic structure, with only minor adjustments in side chains and domain arrangements. In contrast, the compact conformation undergoes a conformational change where the NSH3 domain approaches the SH2 domain, forming a more compact structure with new contacts between these domains, as shown in Figure 4B.The contact map shows that the most significant difference between these two structures is that new contacts have emerged between the CSH3 and SH2 domains due to their more compacted form. In contrast, some contacts between the NSH3 and SH2 domains present in the crystallographic structure are lost due to these changes.

**Figure 4:**
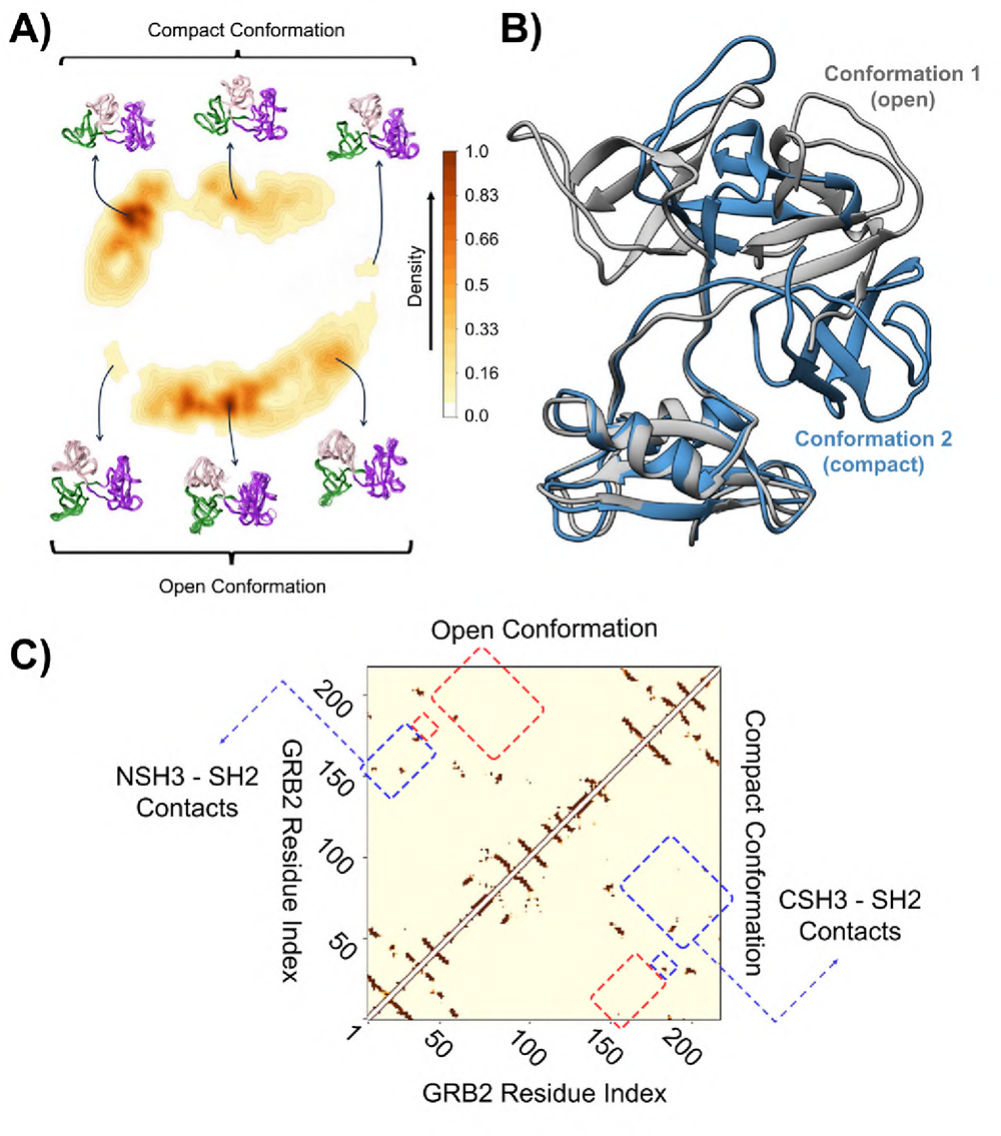
Free energy profile and mean contact map of GRB2. (A) 2D representation of the conformational phase space according to the density of states. The density is computed using a Gaussian Kernel (KDE) and is depicted by overlaying the colormap and contour plot onto the gray ELViM projection dots. (B) The highlighted conformations are superimposed and color-coded: gray represents the open conformation crystallographic structure, and blue represents the compact conformation, with the CSH3 and SH2 domains brought closer together. (C) Mean contact map highlighting the main intraprotein interactions, with the open conformation on the left and the compact conformation on the right. Red highlights indicate the absence of contacts in the conformation, while blue highlights denote the presence of these contacts. It is noted that some interactions between NSH3 and CSH3 present in open conformation are no longer present in compact conformation, and the contacts between CSH3 and SH2 only appear in the compact conformation.

### Monomer-Peptide Complex Molecular Dynamics

We used the experimental information by Liao et al.^24^ and our theoretical data from simulations of the isolated domains (section Selection of Key Peptides) to perform docking of the peptides (S04, S05, S09, and S10) on GRB2 monomers (conformations open and compact – Figure 4). In total, we obtained 8 complexes: S04S10, S05S04, S05S10, and S09S04, in both conformations. The naming follows the pattern of the first peptide binding to the NSH3 domain and the second peptide binding to the CSH3 domain. The S09S10 combination was excluded from the monomer–peptide simulations due to structural limitations highlighted in the reference study.^24^ While each peptide binds effectively to its respective SH3 domain, the short 17-residue linker between them lacks the flexibility needed for simultaneous binding within full-length SOS1, making the S09S10 configuration biologically unlikely. Each of these configurations was selected based on the best interaction energies and further refined by previous data obtained from the MD between domains and peptides, as detailed in section Analysis of SH3/Peptide Complexes.

As shown in Figure 5, we separated the structure of each complex in the first MD frame and after 500 ns of MD, clustered the most representative conformation for each case. This initial result shows that the peptides bound to the NSH3 domain exhibited conformational rearrangement, achieving a preferential structure during MD. This phenomenon is also observed for peptides interacting with the CSH3 domain, although to a lesser extent. Comparing the effects dependent on GRB2 conformations, the results indicate that complexes formed with the compact structure are more stable, with only minor conformational variations in the peptide structures (except for peptide S05 in S05S10). Further analysis revealed that both conformations remain stable even in the presence of peptides, with only minor side chain rearrangements. All these data are graphically reported through RMSD and radius of gyration in Figure S4A,B.

**Figure 5:**
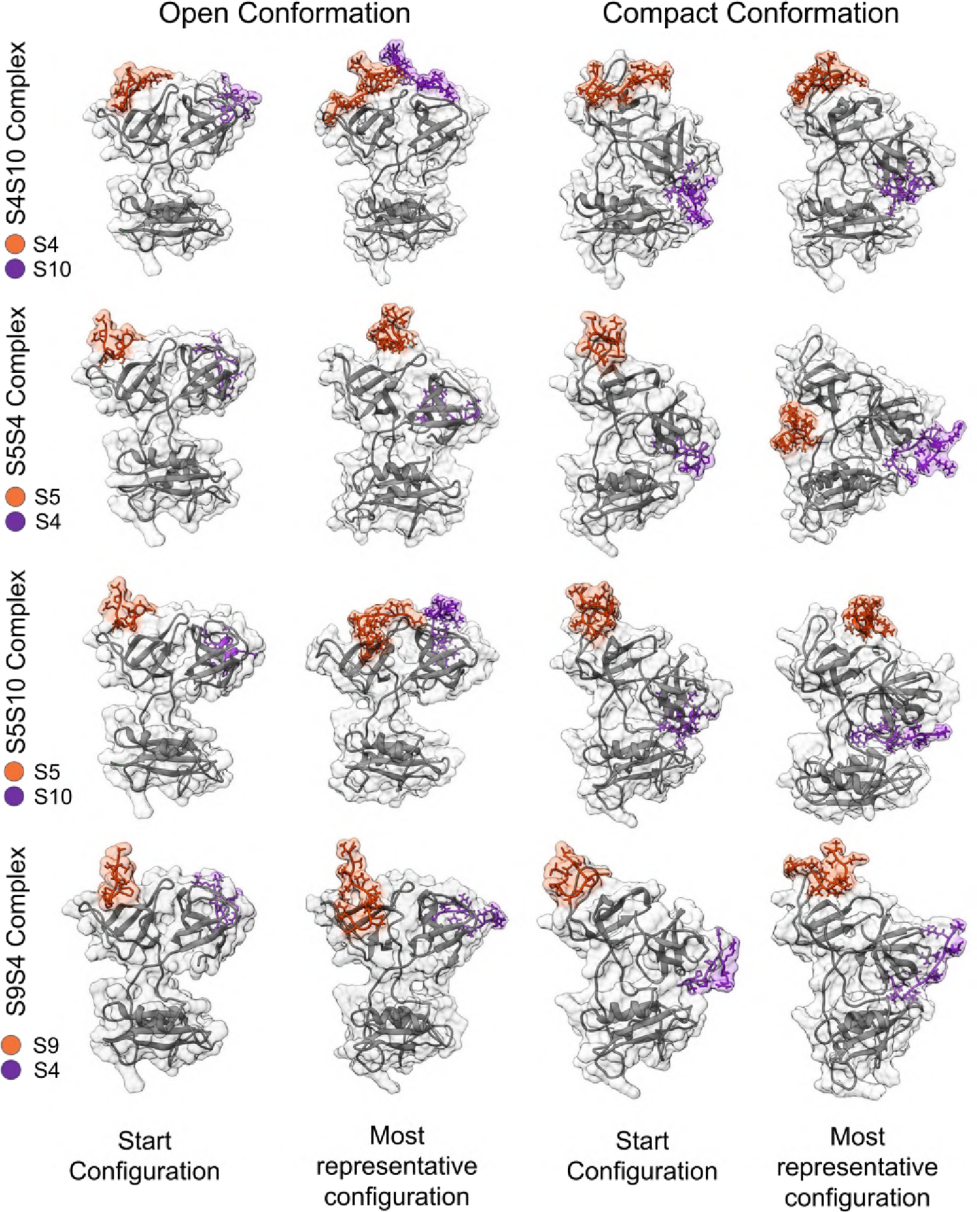
GRB2-SOS1 complex before and after molecular dynamics. We separated the open and compact conformations complexed with peptides (results obtained via molecular docking). Each of these complexes is represented by the initial conformation (structures on the left) and the clustering of the most representative structure of each after 500 ns of molecular dynamics (structures on the right). The peptides are color-coded and correspond to the legend next to the names of the complexes. Overall, all complexes showed some conformational changes to a more stable state.

The association of the S04, S05, and S09 peptides in the NSH3 domain followed as expected from previously obtained data. S04 and S10 follow the PxxPxR and PxxPxK motifs, respectively. This similarity also reflects the stability of the MD simulations, consistently maintaining an equilibrium interface between SOS1-NSH3. The S05 peptide, although also following the PxxPxR motif, is not as stable, a result that corroborates with experimental data.^24^ This difference is largely due to the presence of positively charged residues in S04 and S10, which fit into the binding sites, interacting primarily with phenylalanines, tyrosines, and tryptophans. For CSH3, the S04 and S10 peptides are also stable; however, the new perspective of conformational change shows that the interaction in structure 2, where CSH3 approaches SH2, is more stable. This factor establishes the connection between the binding modes of SOS1 with GRB2 that were not fully elucidated, as the analysis previously focused only on the interaction with the CSH3 domain without considering the presence of the SH2 domain, thus reducing the possibilities to specific peptides (such as S04 and S10).

We characterized the interactions of the peptides in relation to the residues of the GRB2 protein, highlighting those with the greatest influence on complex formation. To achieve this, we calculated the average interaction energy for each site using the MM/GBSA technique, separating them by conformations (open and compact). The results show that for both domains in the open conformation (Figure 6A), the residues Trp36 and Phe47 of the NSH3 domain stand out compared to others. Their nonpolar characteristics and aromatic side chains are crucial factors that allow for optimal peptide accommodation and are responsible for the SOS1-NSH3 complex being more stable compared to SOS1-CSH3. Other residues, such as Lys10, Gln34, Asp45, Glu174 and Asp190, also show significant interaction energy values compared to the overall average and stand out in the interaction. It is noteworthy that, on average, a peptide anchored in the NSH3 domain is influenced by residues from the CSH3 domain and vice versa, highlighting the importance of interdomain communication within GRB2.

**Figure 6:**
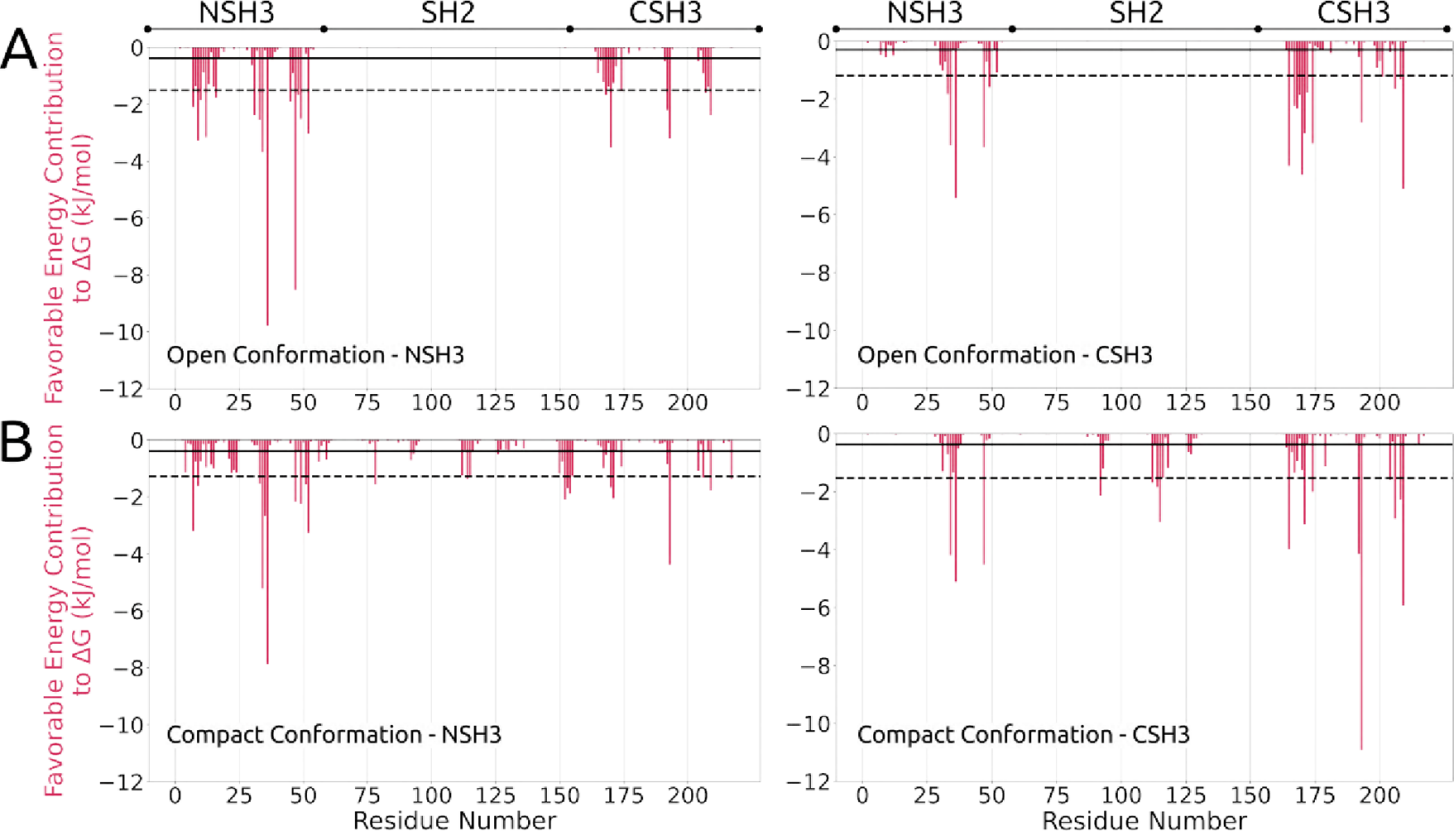
MM/GBSA calculation between GRB2 and SOS1 peptides. Binding free energy by the MM/GBSA method, utilizing only the favorable binding energy (shown in red), for both domain open (A) and domain compact (B) conformation. The left side shows the NSH3 domain, and the right side shows the CSH3 domain. The first cutoff line represents the mean, while the dashed line at the second cutoff point indicates the mean plus standard deviation.

The interactions in the compact conformation yield results similar to the open conformation, with the difference that the key residues obtained are Trp193 and Tyr209, both belonging to the CSH3 domain. This suggests an increase in the stability of the SOS1-CSH3 complex compared to the open conformation. Our main hypothesis remains that plasticity in the arrangements of the GRB2 domains allows for new modes of interaction not observed in the context of isolated domains, potentially influencing the interaction of other peptides and expanding the number of possible fits in the GRB2-SOS1 complex. In this case, the proximity of the CSH3 and SH2 domains led to some residues in the SH2 domain that contributed to the stability of the interaction. Results for unfavorable residues for the interaction were also calculated (see Figure S5). However, compared to favorable ones, their values are minimal, emphasizing the high specificity of the SOS1 peptides for the GRB2 residues.

### Correlated Analyses Reveal SH2 Domain Participation in GRB2 Conformation-Dependent Interactions and Highlight Key Residues for GRB2-SOS1 Interaction

This new perspective led us to investigate the influence of peptide interaction on the GRB2 domains, including the SH2 domain. We calculated RMSF values (Figure 7A) and the persistence of hydrogen bond interactions (Figure 7B). We used ΔRMSF to show the difference between the bound structures and the unbound structure, where values less than zero (ΔRMSF < 0) indicate residues that fluctuated less due to peptide interaction.

**Figure 7:**
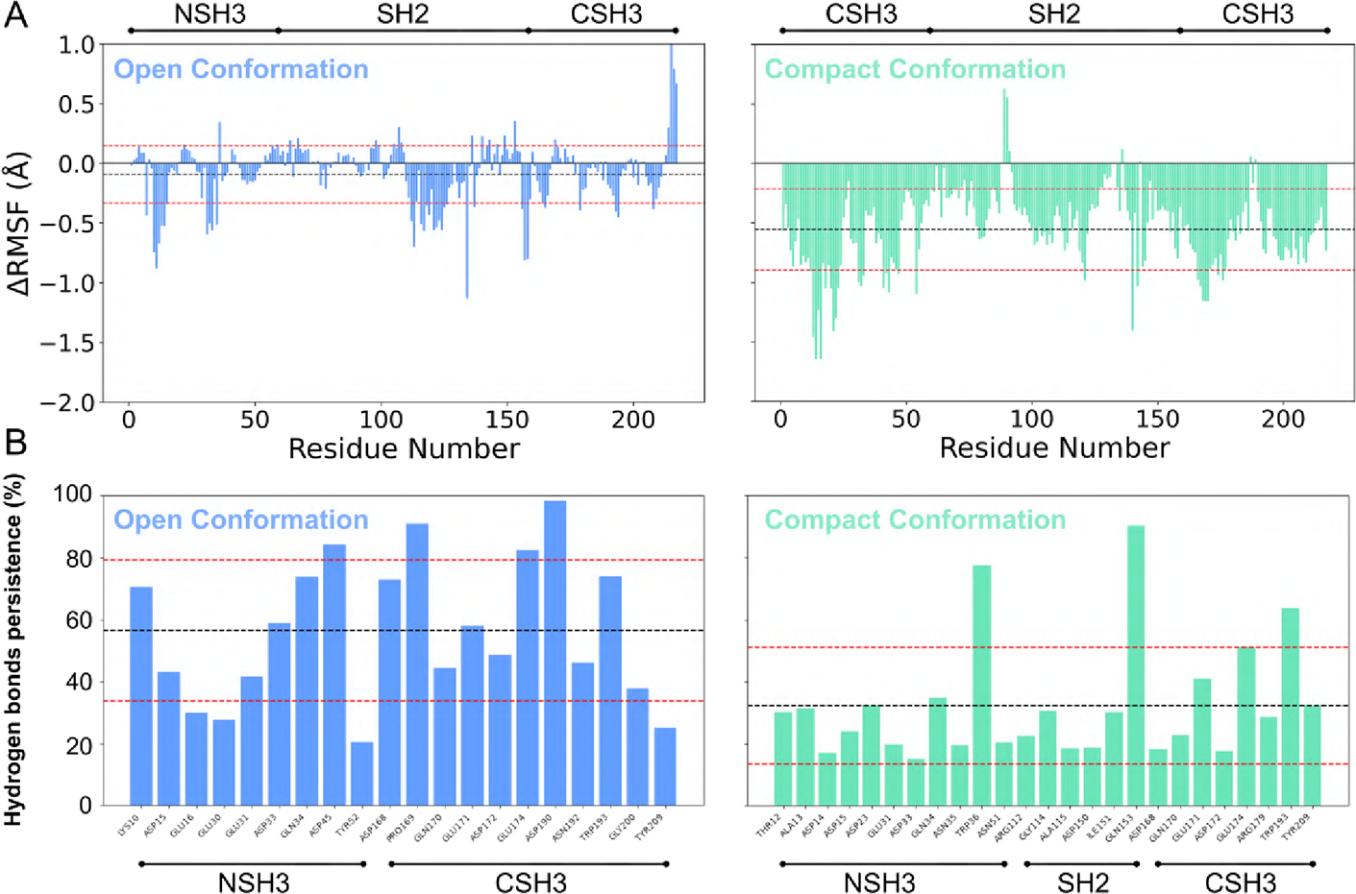
RMSF and hydrogen bonds of monomeric GRB2 complexes and SOS1 peptides. (A) We analyzed the dynamics together, obtaining averages of the values representing the fluctuations of each residue in the open and compact conformations. The RMSF is calculated by the difference between the unbound form and the bound form; thus, negative values represent more stable residues in the presence of the peptide (S04, S05, and S09 are used for NSH3, while S04 and S10 are used for CSH3). It is noted that the compact conformation stabilizes fluctuations in the presence of the peptides. (B) The hydrogen bond interactions follow the same averaging considerations as the RMSF, and the values shown had a persistence cutoff greater than 20%. Noteworthy here is the presence of some direct interactions with the SH2 domain due to the conformational change (compact conformation).

Comparing the two conformations of GRB2, we observed that in open conformation, the effects of fluctuations were subtle, with some specific regions being stabilized due to the interaction. Notably, the SH2 domain had a larger proportion of its residues stabilized. In compact conformation, the reduction in residue fluctuations was more widespread across all three GRB2 domains, indicating that the different structural conformations, due to their flexibility, can modulate the interactions and their effects.

The persistence of hydrogen bonds was calculated to identify which protein residues have direct interactions with the peptides. We considered only hydrogen bonds that persisted for 20% or more of the time during MD simulations. This allowed us to highlight some key residues, such as Asp45, Pro169, Glu174 and Asp190 in the open conformation, and Trp36, Gln153 and Trp193 in the compact conformation. Notably, in the open conformation, although there are no direct hydrogen bond bridges between SH2 and the peptides, other effects were observed, such as differences in RMSF of some SH2 residues. This also underscores the importance of interdomain communication in GRB2.

A combined analysis of hydrogen bonds, favorable energy contributions to ΔGb, and changes in residue fluctuations due to the interaction, covering a broad conformational space derived from eight different 500 ns MD simulations of *GRB*2 – *SOS*1_*PEPTIDES*_ (S04, S05, S09, and S10) complexes in two different conformations (open and compact), allowed us to identify key residues of GRB2 for this interaction. Some of these residues corroborate theoretical docking and dynamics analyses, as well as NMR results determined for the complexes using only the domains.^24–26^ In previous studies, residues Gln34, Trp36 and Trp193 in the loop regions of GRB2’s SH 3s were identified and were also highlighted in the present study as promising for the interaction (Figure 8). Additionally, we emphasize residues Asp33, Pro169, Glu171 and Glu174, which showed significant effects in all computational analyses conducted and, although not directly cited in previous studies, are part of the interaction’s highlighted regions. Equally important, residues Tyr7, Phe9, Lys10, Asp45, Phe47, Phe49, Tyr52, Phe165, Asp168, Gln170 and Asn192 complete the set, allowing us to map the interaction pockets and also demonstrating the importance of GRB2’s structural flexibility for the interactions, with some notable residues belonging to the SH2 domain.

**Figure 8:**
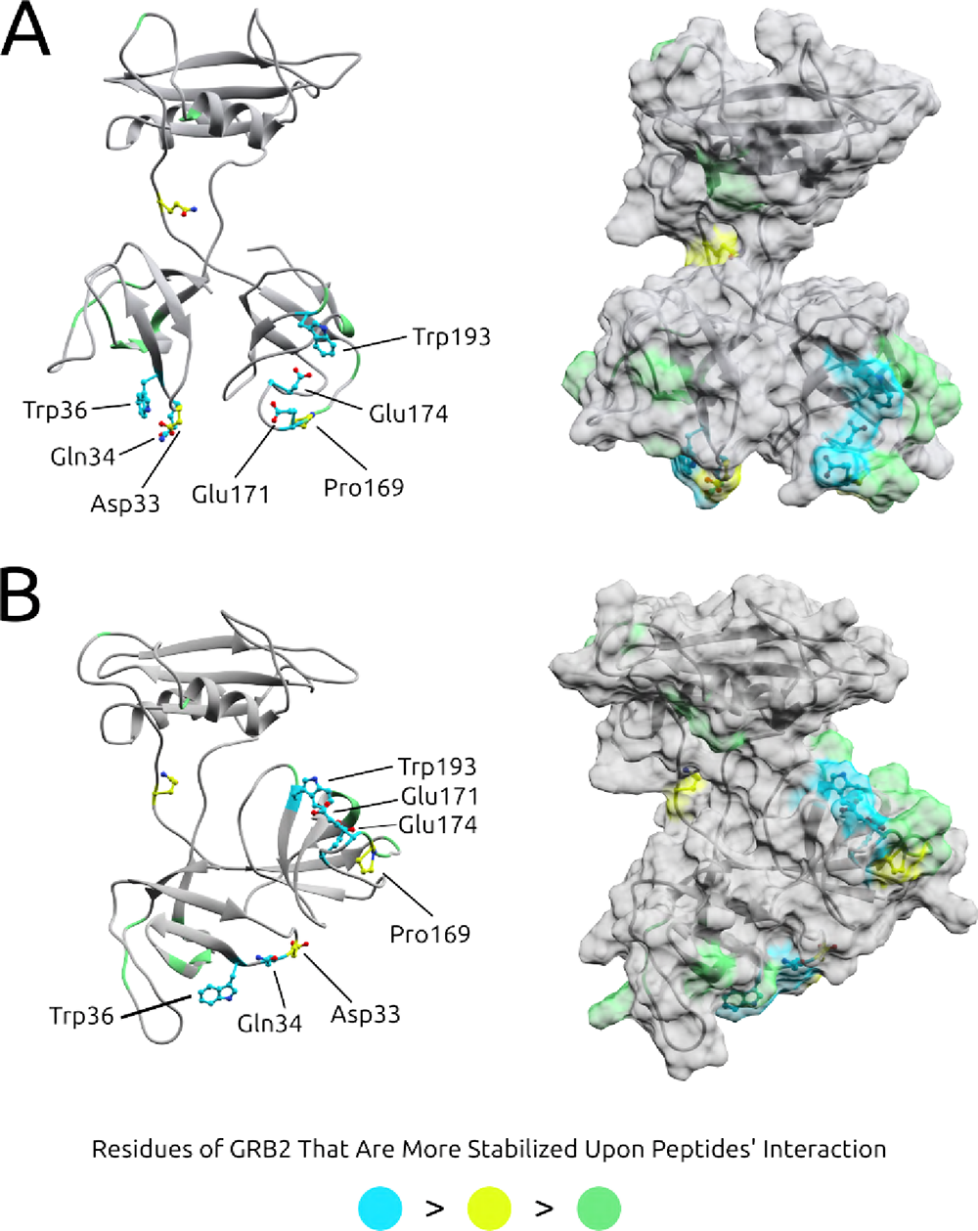
Key residues for the GRB2-SOS1 interaction depicted in (A) open conformations and (B) compact conformation. We separated each of the previous results, counting the key values as well as the number of times these residues appear in the analyses. All key residues are listed in the table, with some highlighted due to their importance in computational analyses, with cyan indicating the most significant, followed by yellow and then green. We compared with previous results and were able to map the key residues for the interaction between GRB2 and SOS1. As a new finding, we show that this interaction is dependent on the configurational form of GRB2, with more compact forms showing more residues stabilizing the interaction, as represented in the image by some green residues in SH2.

## Conclusions

The results presented in this study provide, for the first time, a comprehensive molecular and structural characterization of the interaction between monomeric GRB2 protein and key SOS1 peptides, using computational tools such as modeling, docking, molecular dynamics simulations and dimensional reduction analyzes. Additionally, we characterized these interactions across different structural configurations of GRB2, generating a set of results that encompasses the diversity of possible GRB2 conformations. This interaction is crucial for cellular signaling, playing a fundamental role in signaling pathways such as the MAPK pathway, which regulates cell growth, differentiation, and survival. The interactions of peptides with the NSH3 and CSH3 domains of GRB2 reveal structural stability over 500 ns trajectories. These dynamics were performed for both isolated domain complexes and monomeric GRB2 in two different conformations, characterizing a broad conformational space for the studied system.

From the analyses of these trajectories, we identified that the NSH3 domain acts as a primary interaction site, with a higher number of essential residues for interaction (Asn4, Asp14, Asp15, Asn29, Tyr37, Ala39, Glu40, Leu41), corroborating experimental results from the literature.^24–26,37,39,57^ The analyses of the different conformations of GRB2 highlight their importance for the stabilization of the interaction, emphasizing a interaction between between NSH3 and SH2 that leads to greater stability for the complex, underscoring the importance of interdomain communication in GRB2. Through a broad conformational space exploration and statistical analyses, alongside previous results, we highlighted that Trp193 and Tyr209 play a crucial role within the interaction, offering valuable insights for future studies, such as designing mimetic peptides that could enhance or inhibit the GRB2-SOS1 complex. It is worth noting that this work does not concern the potential competition or preferential binding among peptides, focusing solely on the interaction between the GRB2 and peptides residues. In a broader context, this study contributes a molecular basis for more extensive investigations of the GRB2-SOS1 interaction, moving beyond the minimalist view of interaction with isolated domains to effects arising from the structural complexity of monomeric GRB2. However, it is worth noting that these findings do not correspond to the fully interaction with the full-length SOS1.

## Supplementary Material



## Data Availability

Details for the molecular dynamics simulations and all the analysis protocols are provided in the Materials and Methods sections. The trajectory files, Python scripts, and experimental data are available on Zenodo at the following DOI:10.5281/zenodo.13314538.

## Notes

Table S1, NSH3 and CSH3 domains and their respective peptide complexes; Figure S1, clustering of peptide conformations (S04, S05, S09, and S10) obtained from molecular dynamics simulations under various interaction conditions with the GRB2 protein; Figure S2, clustering of peptide structures (S04, S05, S09, and S10) analyzed using the ELViM methodology; Figure S3, RMSD of the SH3 domains; Figure S4, comparison of the open and compact structures of the GRB2 protein; Figure S5, MM/GBSA calculation between GRB2 and SOS1 peptides (PDF)

## References

[1] Belov A. A., Mohammadi M. (2012). Grb2, a double-edged
sword of receptor tyrosine kinase
signaling. Sci. Signaling.

[2] Tari A. M., Lopez-Berestein G. (2001). GRB2: a pivotal
protein in signal transduction. Semin. Oncol..

[3] Chardin P., Cussac D., Maignan S., Ducruix A. (1995). The GRB2 adaptor. FEBS letters.

[4] Cherfils J., Chardin P. (1999). GEFs: structural basis
for their activation of small
GTP-binding proteins. Trends in biochemical
sciences.

[5] Margolis B., Skolnik E. Y. (1994). Activation of Ras by receptor tyrosine kinases. Journal of the American Society of Nephrology.

[6] Lin C.-W., Nocka L. M., Stinger B. L., DeGrandchamp J. B., Lew L. N., Alvarez S., Phan H. T., Kondo Y., Kuriyan J., Groves J. T. (2022). A two-component
protein condensate
of the EGFR cytoplasmic tail and Grb2 regulates Ras activation by
SOS at the membrane. Proc. Natl. Acad. Sci.
U. S. A..

[7] Lin C.-C., Melo F. A., Ghosh R., Suen K. M., Stagg L. J., Kirkpatrick J., Arold S. T., Ahmed Z., Ladbury J. E. (2012). Inhibition
of basal FGF receptor signaling by dimeric Grb2. Cell.

[8] Ahmed Z., Lin C.-C., Suen K. M., Melo F. A., Levitt J. A., Suhling K., Ladbury J. E. (2013). Grb2 controls phosphorylation of
FGFR2 by inhibiting receptor kinase and Shp2 phosphatase activity. J. Cell Biol..

[9] Ahmed Z., Timsah Z., Suen K. M., Cook N. P., Lee G. R., Lin C.-C., Gagea M., Marti A. A., Ladbury J. E. (2015). Grb2 monomer–dimer equilibrium determines normal
versus oncogenic function. Nat. Commun..

[10] Chardin P., Camonis J. H., Gale N. W., Van Aelst L., Schlessinger J., Wigler M. H., Bar-Sagi D. (1993). Human Sos1:
a guanine
nucleotide exchange factor for Ras that binds to GRB2. Science.

[11] Liao T.-J., Jang H., Fushman D., Nussinov R. (2018). Allosteric KRas4B can
modulate SOS1 fast and slow Ras activation cycles. Biophys. J..

[12] Burotto M., Chiou V. L., Lee J.-M., Kohn E. C. (2014). The MAPK
pathway
across different malignancies: a new perspective. Cancer.

[13] Fruman D.
A., Chiu H., Hopkins B. D., Bagrodia S., Cantley L. C., Abraham R. T. (2017). The PI3K
pathway in human disease. Cell.

[14] Wang D., Liu G., Meng Y., Chen H., Ye Z., Jing J. (2024). The Configuration
of GRB2 in Protein Interaction and Signal Transduction. Biomolecules.

[15] Braicu C., Buse M., Busuioc C., Drula R., Gulei D., Raduly L., Rusu A., Irimie A., Atanasov A. G., Slaby O. (2019). A comprehensive
review on MAPK: a promising therapeutic
target in cancer. Cancers.

[16] Morris V., Kopetz S. (2013). BRAF inhibitors in clinical oncology. F1000Prime Rep..

[17] McNamara C. R., Degterev A. (2011). Small-molecule inhibitors
of the PI3K signaling network. Future medicinal
chemistry.

[18] Lu S., Jang H., Zhang J., Nussinov R. (2016). Inhibitors of Ras–SOS
interactions. ChemMedChem..

[19] Liao T.-J., Jang H., Tsai C.-J., Fushman D., Nussinov R. (2017). The dynamic
mechanism of RASSF5 and MST kinase activation by Ras. Phys. Chem. Chem. Phys..

[20] Ye Y.-B., Lin J.-Y., Chen Q., Liu F., Chen H.-J., Li J.-Y., Liu W.-Q., Garbay C., Vidal M. (2008). The cytotoxicity
of a Grb2-SH3 inhibitor in Bcr-Abl positive K562 cells. biochemical pharmacology.

[21] Yu Y., Nie Y., Feng Q., Qu J., Wang R., Bian L., Xia J. (2017). Targeted covalent inhibition
of Grb2–Sos1 interaction through
proximity-induced conjugation in breast cancer cells. Mol. Pharmaceutics.

[22] Maignan S., Guilloteau J.-P., Fromage N., Arnoux B., Becquart J., Ducruix A. (1995). Crystal structure
of the mammalian Grb2 adaptor. Science.

[23] Dias R. V., Pedro R. P., Sanches M. N., Moreira G. C., Leite V. B., Caruso I. P., de Melo F. A., de Oliveira L. C. (2023). Unveiling
Metastable Ensembles of GRB2 and the Relevance of Interdomain Communication
during Folding. J. Chem. Inf. Model..

[24] Liao T.-J., Jang H., Nussinov R., Fushman D. (2020). High-affinity interactions
of the nSH3/cSH3 domains of Grb2 with the C-terminal proline-rich
domain of SOS1. J. Am. Chem. Soc..

[25] Liao T.-J., Jang H., Fushman D., Nussinov R. (2020). SOS1 interacts with
Grb2 through regions that induce closed nSH3 conformations. J. Chem. Phys..

[26] McDonald C. B., Seldeen K. L., Deegan B. J., Farooq A. (2009). SH3 domains of Grb2
adaptor bind to PXψPXR motifs within the Sos1 nucleotide exchange
factor in a discriminate manner. Biochemistry.

[27] Kessels H. W., Ward A. C., Schumacher T. N. (2002). Specificity
and affinity motifs for
Grb2 SH2-ligand interactions. Proc. Natl. Acad.
Sci. U. S. A..

[28] Simon J. A., Schreiber S. L. (1995). Grb2 SH3 binding to peptides from Sos: evaluation of
a general model for SH3-ligand interactions. Chemistry & biology.

[29] Diop A., Santorelli D., Malagrinò F., Nardella C., Pennacchietti V., Pagano L., Marcocci L., Pietrangeli P., Gianni S., Toto A. (2022). SH2 domains: folding,
binding and
therapeutical approaches. International Journal
of Molecular Sciences.

[30] Kurochkina N., Guha U. (2013). SH3 domains: modules of protein–protein interactions. Biophysical reviews.

[31] Dionne U., Percival L. J., Chartier F. J., Landry C. R., Bisson N. (2022). SRC homology
3 domains: Multifaceted binding modules. Trends
Biochem. Sci..

[32] Pierre S., Coumoul X. (2011). Understanding SOS (son of sevenless). Biochemical pharmacology.

[33] Egan S. E., Giddings B. W., Brooks M. W., Buday L., Sizeland A. M., Weinberg R. A. (1993). Association of Sos
Ras exchange protein with Grb2 is
implicated in tyrosine kinase signal transduction and transformation. Nature.

[34] Li N. a., Batzer A., Daly R., Yajnik V., Skolnik E., Chardin P., Bar-Sagi D., Margolis B., Schlessinger J. (1993). Guanine-nucleotide-releasing
factor hSos1 binds to Grb2 and links receptor tyrosine kinases to
Ras signalling. Nature.

[35] Houtman J. C., Yamaguchi H., Barda-Saad M., Braiman A., Bowden B., Appella E., Schuck P., Samelson L. E. (2006). Oligomerization
of signaling complexes by the multipoint binding of GRB2 to both LAT
and SOS1. Nature structural & molecular
biology.

[36] Bartelt R.
R., Light J., Vacaflores A., Butcher A., Pandian M., Nash P., Houtman J. C. (2015). Regions outside of conserved PxxPxR
motifs drive the high affinity interaction of GRB2 with SH3 domain
ligands. Biochimica et Biophysica Acta (BBA)-Molecular
Cell Research.

[37] McDonald C. B., Seldeen K. L., Deegan B. J., Farooq A. (2008). Structural
basis of
the differential binding of the SH3 domains of Grb2 adaptor to the
guanine nucleotide exchange factor Sos1. Archives
of biochemistry and biophysics.

[38] Yuzawa S., Yokochi M., Hatanaka H., Ogura K., Kataoka M., Miura K.-i., Mandiyan V., Schlessinger J., Inagaki F. (2001). Solution structure of Grb2 reveals extensive flexibility
necessary for target recognition. Journal of
molecular biology.

[39] Kazemein
Jasemi N. S., Herrmann C., Magdalena Estirado E., Gremer L., Willbold D., Brunsveld L., Dvorsky R., Ahmadian M. R. (2021). The intramolecular allostery of GRB2
governing its interaction with SOS1 is modulated by phosphotyrosine
ligands. Biochem. J..

[40] Sethi A., Goldstein B., Gnanakaran S. (2011). Quantifying intramolecular binding
in multivalent interactions: a structure-based synergistic study on
Grb2-Sos1 complex. PLoS computational biology.

[41] Tedesco J. A., Dias R. V., Casteluci G., Pedro R. P., de Oliveira L. C., Caruso Í. P., Melo F. A. (2023). The influence of pH on the structure
and stability of the Grb2 dimer reveals changes in the inter-domain
and molecular interaction: Could it be a modulation mechanism?. Biophys. Chem..

[42] McDonald C. B., Seldeen K. L., Deegan B. J., Lewis M. S., Farooq A. (2008). Grb2 adaptor
undergoes conformational change upon dimerization. Archives of biochemistry and biophysics.

[43] McDonald C. B., El Hokayem J., Zafar N., Balke J. E., Bhat V., Mikles D. C., Deegan B. J., Seldeen K. L., Farooq A. (2013). Allostery
mediates ligand binding to Grb2 adaptor in a mutually exclusive manner. Journal of molecular recognition.

[44] Yang J., Yan R., Roy A., Xu D., Poisson J., Zhang Y. (2015). The I-TASSER
Suite: protein structure and function prediction. Nat. Methods.

[45] Abraham M.
J., Murtola T., Schulz R., Páll S., Smith J. C., Hess B., Lindahl E. (2015). GROMACS: High performance
molecular simulations through multi-level parallelism from laptops
to supercomputers. SoftwareX.

[46] Case D. A., Aktulga H. M., Belfon K., Cerutti D. S., Cisneros G. A., Cruzeiro V. W. D., Forouzesh N., Giese T. J., Götz A. W., Gohlke H. (2023). AmberTools. J. Chem. Inf. Model..

[47] Strodel B. (2021). Amyloid aggregation
simulations: challenges, advances and perspectives. Curr. Opin. Struct. Biol..

[48] Carballo-Pacheco M., Strodel B. (2017). Comparison of force
fields for Alzheimer’s A:
A case study for intrinsically disordered proteins. Protein science.

[49] Hornak V., Abel R., Okur A., Strockbine B., Roitberg A., Simmerling C. (2006). Comparison of multiple Amber force
fields and development of improved protein backbone parameters. Proteins: Struct., Funct., Bioinf..

[50] Bussi G., Donadio D., Parrinello M. (2007). Canonical
sampling through velocity
rescaling. J. Chem. Phys..

[51] Darden T., York D., Pedersen L. (1993). Particle mesh Ewald:
An N log (N) method for Ewald sums in large systems. J. Chem. Phys..

[52] Viegas R. G., Martins I. B., Sanches M. N., Oliveira Junior A. B., Camargo J. B. d., Paulovich F. V., Leite V. B. (2024). ELViM: Exploring
Biomolecular Energy Landscapes through Multidimensional Visualization. J. Chem. Inf. Model..

[53] Oliveira A. B., Yang H., Whitford P. C., Leite V. B. (2019). Distinguishing
biomolecular pathways and metastable states. J. Chem. Theory Comput..

[54] Lamiable A., Thévenet P., Rey J., Vavrusa M., Derreumaux P., Tufféry P. (2016). PEP-FOLD3: faster de novo structure
prediction for
linear peptides in solution and in complex. Nucleic acids research.

[55] Dominguez C., Boelens R., Bonvin A. M. (2003). HADDOCK: a protein- protein docking
approach based on biochemical or biophysical information. J. Am. Chem. Soc..

[56] Valdés-Tresanco M. S., Valdés-Tresanco M. E., Valiente P. A., Moreno E. (2021). gmx_MMPBSA:
a new tool to perform end-state free energy calculations with GROMACS. J. Chem. Theory Comput..

[57] Jasemi N. S. K., Ahmadian M. R. (2022). Allosteric regulation of GRB2 modulates
RAS activation. Small GTPases.

