# Supplementary material for: Conformational Flexibility of GRB2 as a Key Factor in the Stability and Regulation of Its Interaction with SOS1
Source: ACS Omega. 2025 Jun 30;10(27):29119–30. doi: 10.1021/acsomega.5c01677 (PMC12268742; doi:10.1021/acsomega.5c01677)
Supplement: Supplementary file 1 [file ao5c01677_si_001.pdf]

SI - Conformational Flexibility of GRB2 as a Key Factor in the Stability and Regulation of Its Interaction with SOS1

Renan P. Pedro,[†] Raphael V. R. Dias,[*,†] Ingrid B. S. Martins,[†] Murilo N. Sanches,[†] João V. Piloto,[†] Icaro P. Caruso,[†] Vitor B. P. Leite,[†,‡] and Fernando A. de Melo[*,†]

†*Department of Physics, São Paulo State University (UNESP), Institute of Biosciences, Humanities and Exact Sciences, São José do Rio Preto, SP, 15054-000, Brazil*

‡*Institute of Chemistry, São Paulo State University (UNESP), Araraquara, SP, 14800-060, Brazil*

E-mail: rvr.dias@unesp.br; fernando.melo@unesp.br

Table S1: NSH3 and CSH3 domains and their respective peptide complexes. The NSH3 domain forms complexes with peptides S04, S05, and S09, while the CSH3 domain interacts with peptides S04 and S10.

Domain	Peptides
**NSH3**	S04
	S05
	S09
**CSH3**	S04
	S10

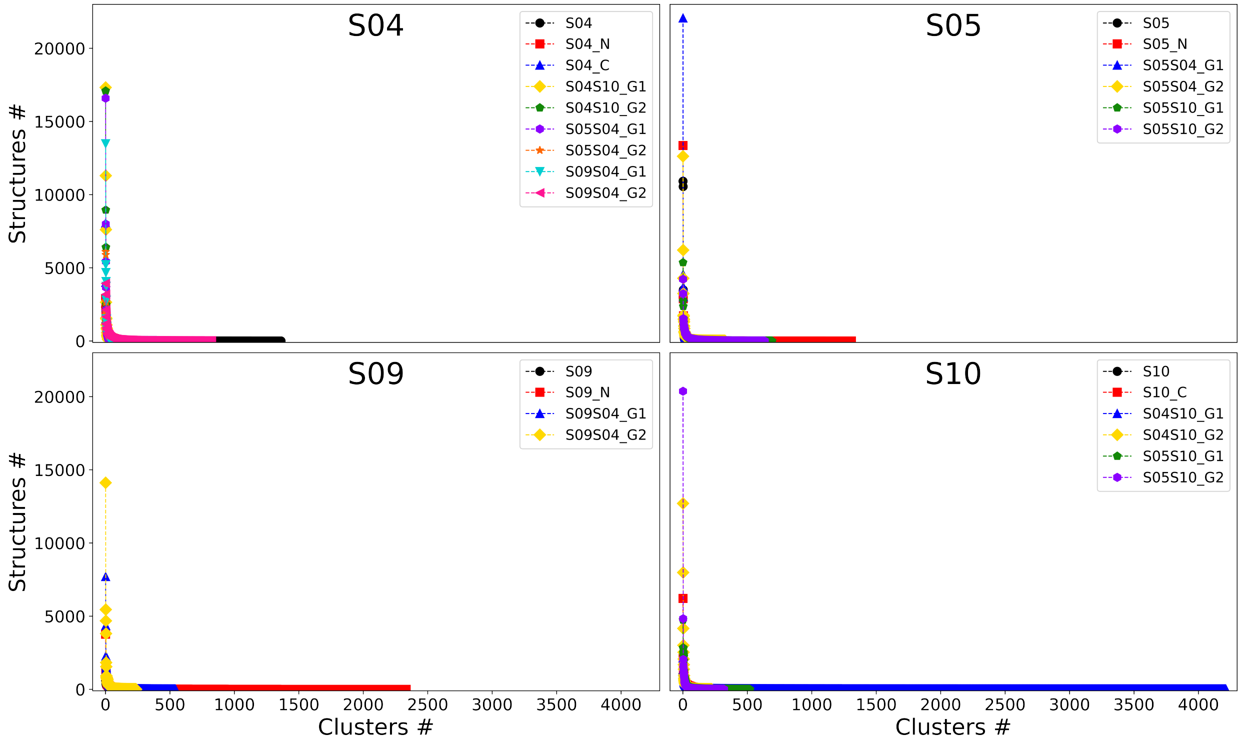

Figure S1: Clustering of peptide conformations (S04, S05, S09, and S10) obtained from molecular dynamics simulations under various interaction conditions with the GRB2 protein. Graph 1 (S04): Clustering of peptide S04 under the following conditions: (1) simulation of the isolated peptide; (2) interaction with the NSH3 domain; (3) interaction with the CSH3 domain; (4-5) interaction with the NSH3 domain (conformations 1 and 2) while the CSH3 domain interacts with peptide S10; (6-7) interaction with the CSH3 domain (conformations 1 and 2) while the NSH3 domain interacts with peptide S05; (8-9) interaction with the CSH3 domain (conformations 1 and 2) while the NSH3 domain interacts with peptide S09. Graph 2 (S05): Clustering of peptide S05 under the following conditions: (1) simulation of the isolated peptide; (2) interaction with the NSH3 domain; (3-4) interaction with the NSH3 domain (conformations 1 and 2) while the CSH3 domain interacts with peptide S04; (5-6) interaction with the NSH3 domain (conformations 1 and 2) while the CSH3 domain interacts with peptide S10. Graph 3 (S09): Clustering of peptide S09 under the following conditions: (1) simulation of the isolated peptide; (2) interaction with the NSH3 domain; (3-4) interaction with the NSH3 domain (conformations 1 and 2) while the CSH3 domain interacts with peptide S04. Graph 4 (S10): Clustering of peptide S10 under the following conditions: (1) simulation of the isolated peptide; (2) interaction with the CSH3 domain; (3-4) interaction with the CSH3 domain (conformations 1 and 2) while the NSH3 domain interacts with peptide S04; (5-6) interaction with the CSH3 domain (conformations 1 and 2) while the NSH3 domain interacts with peptide S05.

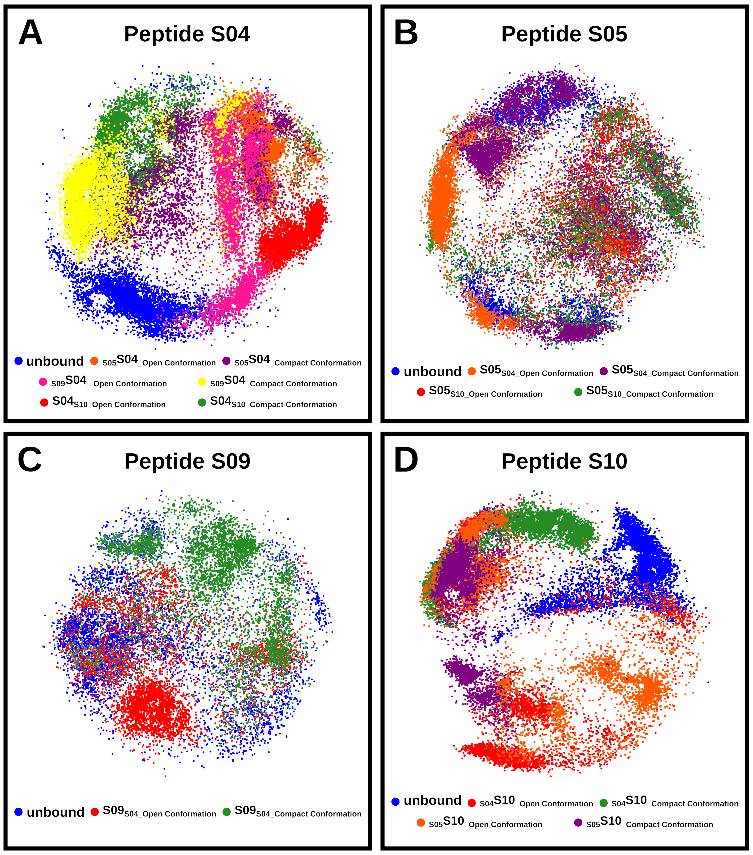

Figure S2: Clustering of peptide structures (S04, S05, S09, and S10) analyzed using the ELViM methodology. (A) Sampling of peptide S04 in its unbound (represented in blue) and bound forms. The interactions examined include: 1) S04 interacting with the NSH3 domain, while peptide S10 interacts with the CSH3 domain (conformations open and compact, represented in red and green, respectively); 2) S04 interacting with the CSH3 domain, with peptide S09 simultaneously interacting with the NSH3 domain (open conformation in pink and compact conformation in yellow); 3) S04 interacting with the CSH3 domain, while peptide S05 interacts with the NSH3 domain (open conformation in red and compact conformation in green). (B) Sampling of peptide S05 in its unbound (blue) and bound forms. The evaluated interactions are: 1) S05 interacting with the NSH3 domain, while peptide S04 interacts with the CSH3 domain (open conformation in orange and compact conformation in purple); 2) S05 interacting with the NSH3 domain, while peptide S10 interacts with the CSH3 domain (open conformation in red and compact conformation in green). (C) Sampling of peptide S09 in its unbound (blue) and bound forms, showing: 1) S09 interacting with the NSH3 domain, while peptide S04 interacts with the CSH3 domain (open conformation in red and compact conformation in green). (D) Sampling of peptide S10 in its unbound (blue) and bound forms, with the interactions: 1) S10 interacting with the CSH3 domain, while peptide S04 interacts with the NSH3 domain (open conformation in red and compact conformation in green); 2) S10 interacting with the CSH3 domain, while peptide S05 interacts with the NSH3 domain (open conformation in orange and compact conformation in purple). The results indicate that peptides S04 and S10 form well-defined clusters, regardless of the presence or absence of ligands, suggesting higher structural stability. In contrast, peptides S05 and S09 exhibit greater conformational variability in their unbound forms, adopting multiple possible conformations. Interaction with the GRB2 protein appears to stabilize certain conformations, particularly for peptides S05 and S09.

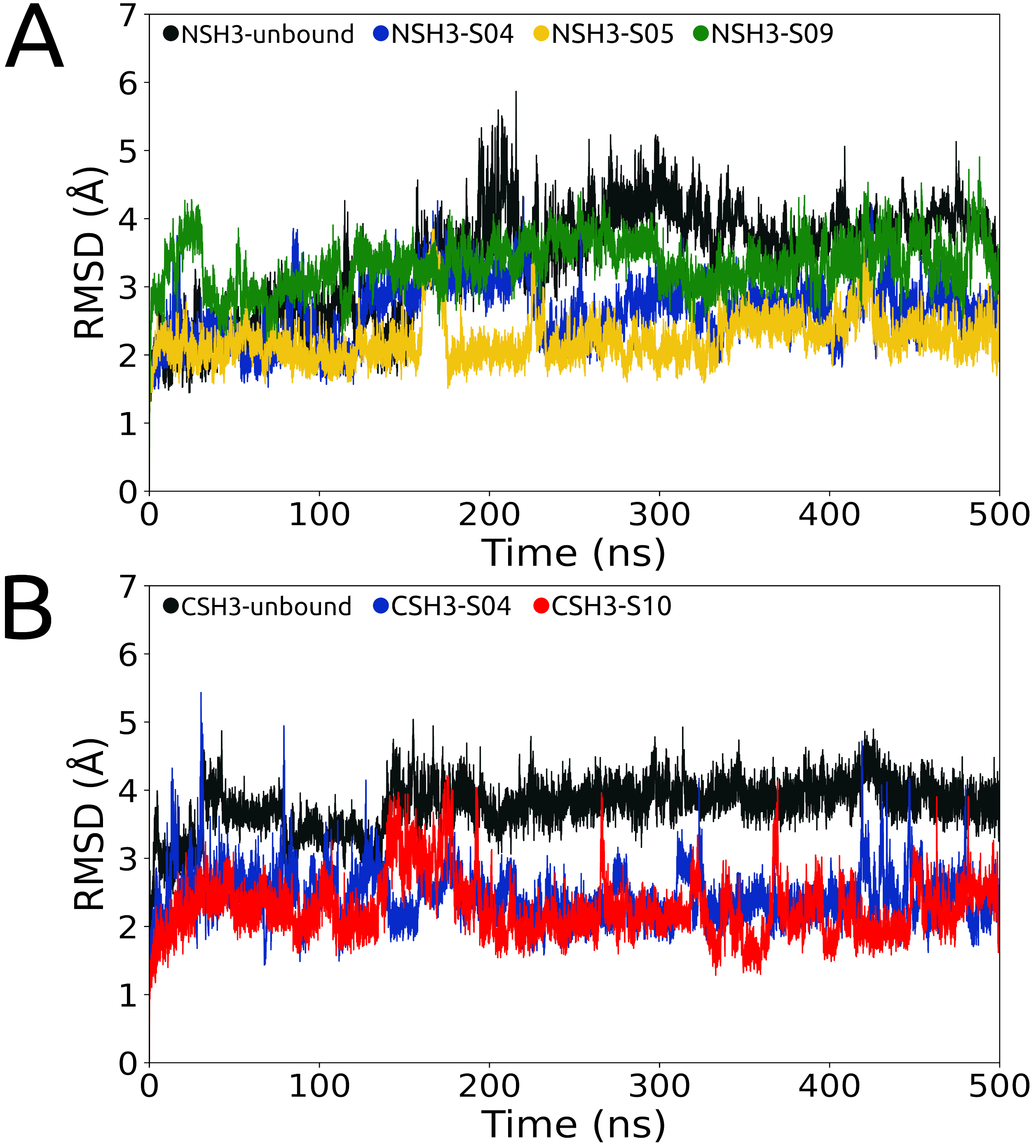

Figure S3: RMSD of the SH3 Domains. (A) In black, the unbound NSH3; in blue, the NSH3 interacting with S04; in yellow, the NSH3 interacting with S05; and in green, the NSH3 interacting with S09. (B) In black, the unbound CSH3; in blue, the CSH3 interacting with S04; and in red, the CSH3 interacting with S10.

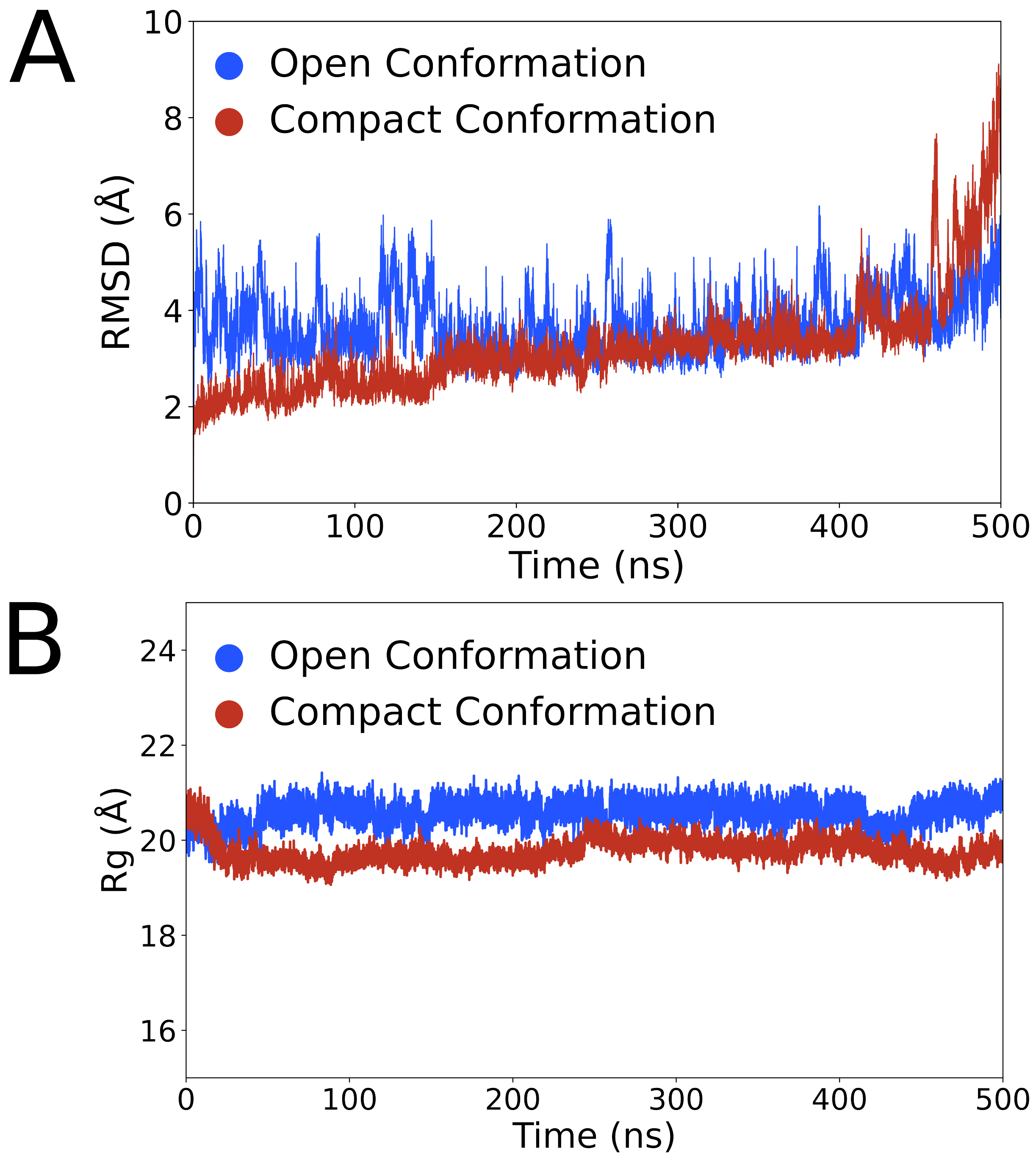

Figure S4: Comparison of the open and compact Structures of the GRB2 Protein. (A) Depicts the RMSD between the structures. (B) Illustrates the radius of gyration of the same structures. The color representations distinguish open conformation in blue and compact conformation in red.

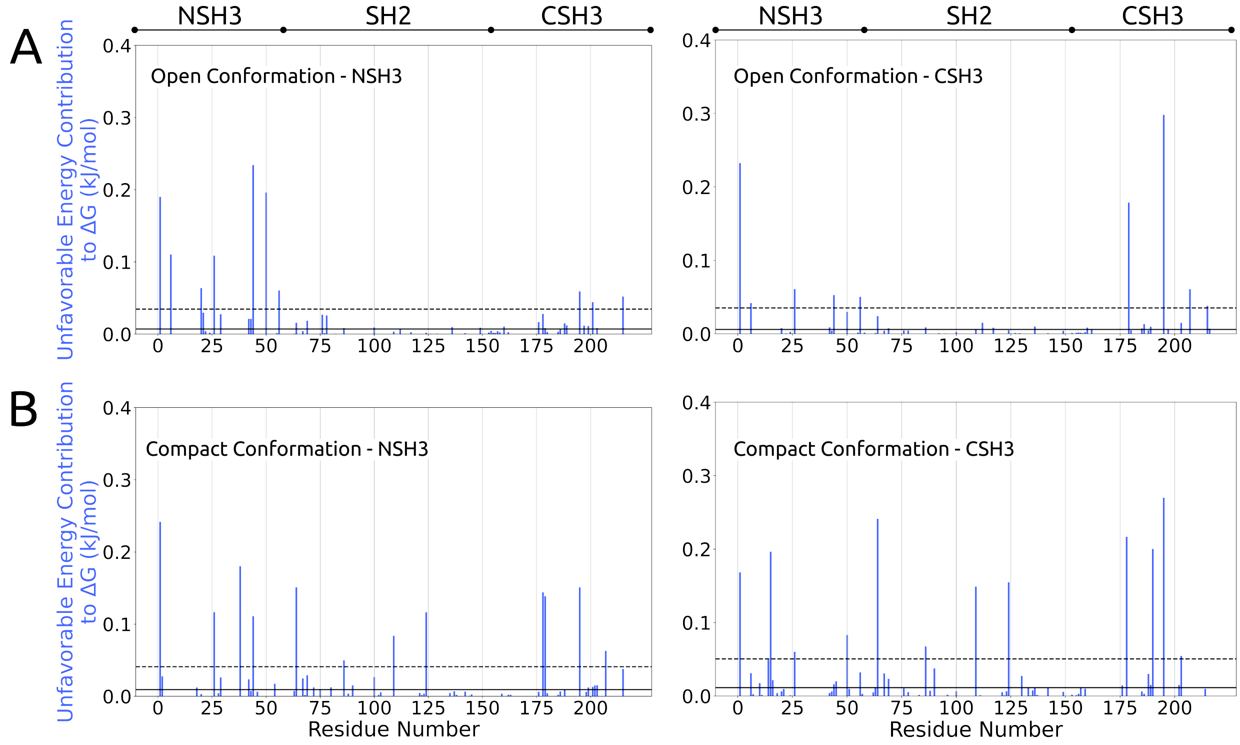

Figure S5: MM/GBSA Calculation Between GRB2 and SOS1 Peptides. Binding free energy by the MM/GBSA method, utilizing only the unfavorable binding energy (shown in blue), for both domain open conformation (A) and domain compact conformation (B). The left side shows the NSH3 domain, and the right side shows the CSH3 domain. The first cutoff line represents the mean, while the dashed line at the second cutoff point indicates the mean plus standard deviation.
